# Adjustable Male Sling for The Treatment of Postprostatectomy Stress Urinary Incontinence: Intermediate-Term Follow-Up Results

**DOI:** 10.7759/cureus.43280

**Published:** 2023-08-10

**Authors:** Fatih Yanaral, Mehmet Hamza Gültekin, Ahmet Halis, Fatih Akbulut, Omer Sarilar, Faruk Ozgor

**Affiliations:** 1 Urology, Haseki Education and Research Hospital, Istanbul, TUR

**Keywords:** prostate, stress urinary incontinence, prostate surgery, male urinary incontinence, adjustable male sling

## Abstract

Objective

To evaluate the outcomes of adjustable male sling (Argus®) implantation in the management of post-prostatectomy incontinence (PPI) with intermediate-term follow-up results.

Materials and methods

The data on adjustable male sling surgery between September 2015 and September 2020 were retrospectively analyzed. Patients were preoperatively evaluated with a voiding diary, 24-hour pad test, and validated questionnaire. Functional outcomes were also evaluated using 24-hour pad requirement and pad weight, and the International Consultation on Incontinence (ICIQ‐SF) score.

Results

A total of 16 patients (eight having undergone the transurethral resection of the prostate [TUR-P] and eight radical prostatectomy [RP]) were enrolled in the study. Thirteen patients had moderate (81.25%) PPI, and three patients (18.75%) had severe PPI. With the mean follow-up of 36.9±14.3 months, nine patients (56.2%) were noted as cured and four (25%) as improved, with an overall success rate of 81.2%. At the last follow-up visit, the median number of pads used per day decreased from 3.5 to 1, and the 24-hour pad test result decreased from 300 to 50 gr (p < 0.001 and p < 0.001, respectively). The ICIQ-SF score decreased from the initial mean of 15.8 ± 2.3 to 7.1 ± 6.6 (p < 0.001). When the outcomes were compared according to the etiology, there was no statistically significant difference (p = 0.522).

Conclusions

Male sling surgery can be performed safely in patients with moderate and severe stress urinary incontinence with low complication and high success rates. The results of TUR-P-related PPI are similar to those of surgery performed due to the etiology of RP.

## Introduction

Stress urinary incontinence (SUI) is a bothersome complication of prostate surgery and has a negative impact on health-related quality of life. The SUI rates vary widely from 11% to 69% after radical prostatectomy (RP) and 1% to 5% after the transurethral resection of the prostate (TURP) [1,2]. The initial management of post-prostatectomy urinary incontinence (PPI) usually consists of conservative therapies, such as medication, pelvic floor exercises, and behavioral therapy. If SUI persists after six to 12 months of conservative treatment, a surgical approach is recommended [3]. Two major surgical options for male SUI are an artificial urinary sphincter (AUS) and a male sling. In addition to the high success rates, AUS has some potential complications, such as infection, urethral atrophy or erosion, and mechanical failure and it requires revision surgery at a rate of up to 30% [4]. Over the last decade, male slings have gained popularity due to the need for a less invasive and risky alternative to AUS [5].

Male sling surgery is based on the concept of urethral support and external urethral compression. A male sling is generally placed under the bulbar urethra using a retropubic or transobturator technique. It is generally evaluated under two groups as adjustable and fixed. Adjustable slings have been developed in order to overcome the potential problems of excessive or insufficient correction of continence [5]. The manipulation of the scrotal pump is not necessary before micturition in male slings, which may be a reason for their preference by patients [5]. One of the most commonly used adjustable male sling is Argus® (Promedon, Cordoba, Argentina). In the literature, the use of Argus® has been reported in many different situations, such as post-TUR-P and following pelvic radiation therapy. However, these different situations constitute a small portion of the general patient population and cannot be generalized to the whole population. There is also no comparative study investigating the postoperative outcomes between SUI after RP and after TUR-P.

In this study, we aimed to evaluate the outcomes of adjustable male sling (Argus®) implantation in the management of PPI based on intermediate-term follow-up results. We consider that our results provide a more objective evaluation of the surgical outcomes of this condition since we had an equal number of patients that developed SUI after TUR-P and RP.

## Materials and methods

Patient population

This study was approved by the institutional ethics committee (approval number: 2020-90) and was conducted in accordance with the Declaration of Helsinki Ethical Principles and Good Clinical Practices for Medical Research Involving Human Subjects.

The records of patients who had been treated with adjustable bulbourethral slings (Argus®) for SUI following prostatic surgery in our clinic between September 2015 and September 2020 were retrospectively analyzed. The patients’ demographic data, preoperative parameters, and postoperative outcomes were recorded prospectively. The inclusion criterion was having post-prostatectomy SUI for at least the past 12 months despite conservative treatment. Patients with a history of pelvic radiotherapy, previous surgery for SUI, mild SUI, untreated urethral stricture or bladder neck contracture, urge, and mixed urinary incontinence due to detrusor overactivity were excluded from the study.

Before the operation, all patients were evaluated in terms of their medical history, physical examination, voiding diary, 24-hour pad test, ultrasonography, urethrocystoscopy, urodynamic analysis findings, and self-administered questionnaire of the validated International Consultation on Incontinence Questionnaire short form (ICIQ-SF) [6]. Urinary incontinence was classified according to the results of the 24-hour pad test as follows: mild incontinence if the patient required one to two pads per day, moderate incontinence if three to five pads per day, and severe incontinence if more than five pads per day. The patients with moderate and severe PPI had undergone male sling surgery; however, none of the patients with mild PPI had undergone this surgery.

Surgical technique

The adjustable Argus® system comprises a silicone foam pad, ribbed silicone columns, and tension-adjusting silicone washers. All patients had sterile urine cultures prior to surgery. The Argus male sling was implanted using the standardized surgical technique described by Romano et al. through the perineal approach [7]. Using needles, the sling was passed through the perineum to the suprapubic region. A silicone foam pad was used to provide bulbar urethral compression by keeping the bulbocavernosus muscles in place. By adjusting the two silicone washers placed on the rectus fascia, the amount of urethral compression was regulated and maintained. The retrograde urethral pressure was controlled by cystoscopy and adjusted to 35-45 cm H20. The radio-opaque pad and washers allow the evaluation of the male sling and its components by radiography during the follow-up. A urethral catheter was inserted and maintained for 24 hours, and the patients were generally discharged on the following day when they were able to urinate on their own.

Follow-up

The follow-up evaluations of the patients were performed at the postoperative third, 12th, 18th, and 36th months and annually thereafter, which included uroflowmetry and post-void residual urine measurements, 24-hour pad test, and the ICIQ-SF score. Complications were categorized and recorded according to the Clavien-Dindo classification [8]. Additionally, the patients were directed to the following three questions, which are routinely used in our daily practice, to evaluate their thoughts concerning the results: Considering your present condition, would you undergo the same surgery again? Are you satisfied with the results? Would you recommend the same surgery to a friend? To respond to these questions, the patients were given three options: yes, somewhat, and no.

The treatment was defined as successful if the patient was cured (≤ 1 pad/day) or improved (not cured but a ≥ 50% reduction in the number of pads required per day). Treatment failure was defined as the absence of a cure or improvement. As a secondary analysis, we compared the variables associated with the success or failure of the procedure [9,10].

Statistics

All statistical analyses were performed using SPSS (IBM Corp. Released 2011. IBM SPSS Statistics for Windows, Version 20.0. Armonk, NY: IBM Corp). The compliance of data to the normal distribution curve was evaluated by the Shapiro-Wilk test. Student’s t-test or the Mann-Whitney U-test was used to assess the baseline variables between the failure and success groups, depending on whether the statistical hypotheses were fulfilled. The Wilcoxon test and the paired-sample t-test were used to evaluate the changes between the baseline and the follow-up data of the patients. The chi-square or Fisher’s exact test was used to compare the categorical variables between the groups. A p of < 0.05 was considered statistically significant.

## Results

Sixteen patients with a mean age of 64.5 ± 6.8 years were included in the study. The baseline characteristics of the patients are given in Table 1. The primary etiology of PPI was RP in eight patients (50%) and TURP in the remaining eight patients (50%). The median number of pads and pad weight per day were 3.5 (range, 3 to 7) and 300 (range, 120 to 800), respectively. The mean ICIQ-SF score and follow-up time were 15.8 ± 2.3 and 36.9 ± 14.3 months, respectively (Table 1). The vast majority of the patients had moderate (81.25%) PPI, and three (18.75%) had severe PPI. The mean operative duration was 107.5 ± 26.7 minutes.

**Table 1:** Patient characteristics ICIQ-SF: International Consultation on Incontinence Questionnaire Short Form, TUR: Transurethral resection

		Variables	
Age (years)		median (range)	64.5 ± 6.8
Etiology	TUR Prostatectomy	n (%)	8 (50%)
	Radical Prostatectomy	n (%)	8 (50%)
Number of pads used per day		median (range)	3.5 (3-7)
Pad weight in 24 hours (gr)		median (range)	300 (120-800)
ICIQ-SF		mean ± standard deviation	15.8 ± 2.3
Follow-up time (month)		mean ± standard deviation	36.9 ± 14.3

**Figure 1:**
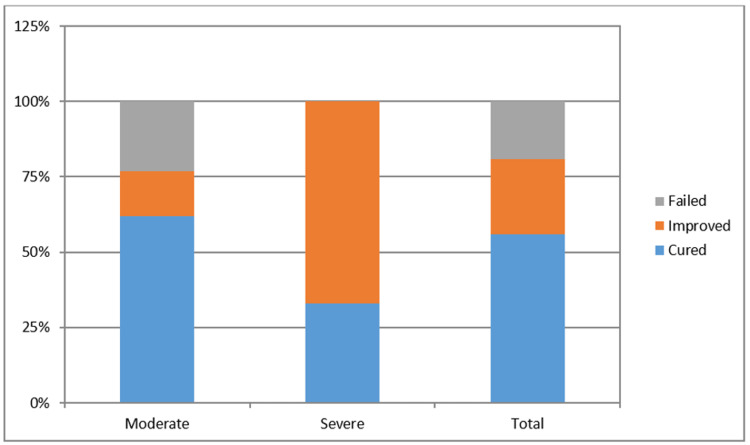
The success rates per degree of incontinence

In one case where the first operation failed, incontinence was improved after one adjustment. In the last follow-up visit, nine patients (56.2%) were noted as cured and four patients (25%) as improved, with an overall success rate of 81.2%. The treatment failed in three patients (18.8%). When we examined patients in whom the male sling surgery failed, we saw that in one patient, urethral stricture was developed after surgery which might be the reason for the failure. In other patients, there was no obvious cause for this condition. The median number of pads used per day, which was 3.5 (range, 3-7) before surgery, decreased to 1 (range, 0-3) at the last follow-up visit (p < 0.001). Urine loss in the 24-hour pad test decreased from 300 (range 120-800) gr to 50 (range, 0-200) gr (p < 0.001). The ICIQ-SF score decreased from the initial mean of 15.8 ± 2.3 to 7.1 ± 6.6 (p < 0.001) (Table 2). The outcomes according to the degree of incontinence are shown in Figure 1.

**Table 2:** Comparison of the baseline parameters with the outcomes of the Argus sling ‡ Success = Cured + Improved, Statistical analysis by the Wilcoxon^a^ test and the paired samples t-test^b^  ICIQ-SF: International Consultation on Incontinence Questionnaire Short Form

		Variables	Baseline	Postoperative last follow-up	p
Number of pads (24 hours)		median (range)	3.5 (3-7)	1 (0-3)	< 0.001^a^
Pad weight in 24 hours (gr)		median (range)	300 (120-800)	50 (0-200)	< 0.001^a^
ICIQ-SF		mean ± standard deviation	15.8 ± 2.3	7.1 ± 6.6	< 0.001^b^
Overall Outcome	Cured	n (%)		9 (56.2%)	
	Improved	n (%)		4 (25%)	
	Success^‡ ^	n (%)		13 (81.2%)	
	Failed	n (%)		3 (18.8%)	

When the patient characteristics were compared according to the outcomes, the median age, etiology, median number of pads used per day, median pad weight within 24 hours, and median ICIQ-SF score were similar between the failure and success groups (p = 0.057, p = 0.522, p = 0.942, p = 0.611 and p = 0.364, respectively) (Table 3). The patients’ thoughts concerning the results are shown in Table 4. When subgroup analysis was performed according to the outcome of surgery, the patients with unfavorable outcomes were more dissatisfied at a statistically significant level (p = 0.018).

**Table 3:** Comparison of the parameters of the patients with treatment failure and success Statistical analysis by the Mann Whitney U^a^ and Fisher’s exact^b^ tests, ICIQ-SF: International Consultation on Incontinence Questionnaire Short Form, TUR: Transurethral resection

		Variables	Failure (n = 3)	Success (n = 13)	p
Age (years)		median (range)	60 (55-65)	65 (55-77)	0.189^a^
Etiology	TUR Prostatectomy	n (%)	2 (25%)	6 (75%)	0.50^b^
	Radical Prostatectomy	n (%)	1 (12.5%)	7 (87.5%)	
Number of pads used per day		median (range)	4 (3-5)	3 (3-7)	0.942^ a^
Pad weight in 24 hours		median (range)	320 (220-380)	300 (120-800)	0.611^ a^
ICIQ-SF		median (range)	17 (15-17)	15 (11-21)	0.364^ a^
Severity of incontinence	Moderate	n (%)	3 (23.1%)	10 (76.9%)	0.511^b^
	Severe	n (%)	0 (0%)	3 (100%)	

**Table 4:** Patients’ thoughts concerning the treatment results

	Variables	Yes	Somewhat	No
Would you undergo the same surgery again?	n (%)	11 (68.75%)	2 (12.5%)	3 (18.75%)
Are you satisfied with the results?	n (%)	11 (68.75%)	2 (12.5%)	3 (18.7%)
Would you recommend this surgery to a friend?	n (%)	11 (68.75%)	2 (12.5%)	3 (18.7%)

During the postoperative follow-up, a total of three (18.8%) complications were recorded. Two patients (one after TURP, one after RP) with urinary retention due to bladder neck and urethral stricture were treated endoscopically, and one patient (after RP) with skin erosion was treated with surgical debridement and primary wound closure (Clavien-Dindo grade IIIa complications). No total sling explanation was required. Due to the low number of complications, we couldn’t compare the relationship between complications and other parameters.

## Discussion

The present study investigated the intermediate outcome of adjustable MS for the treatment of male SUI in clinical daily practice. We found that the overall success rate was 81.2% (56.2% cured, 25% improved) over a mean follow-up of 36.9 ± 14.3 months. If we focus on the current literature in terms of the treatment of patients with moderate to severe PPI, our results are comparable. In a recent multi-center study with the largest series of 182 cases in which this method was applied, the success rates were 78% and 70% in patients with moderate and severe incontinence, respectively [9]. When only the results of patients with moderate and severe incontinence were taken into consideration, as in our study, the cure rate is calculated as 29%, the improvement rate as 45%, and the success rate as 74%. In another article reporting the midterm outcomes of a large number of cases, the moderate PPI group had a 67% success rate (53% cured, 14% improved) while the severe PPI group had a success rate of 67% (46% cured, 21% improved) [10]. Although these results are very similar to our findings, ours are slightly better.

Age, pre-existing lower urinary tract symptoms, TURP before RP, prostate size, and body mass index were the preoperative factors that were most related to PPI [11]. Higher rates of urinary incontinence were seen in cases where the urethral sphincter, pelvic floor structures, or their innervation was damaged during RP. In a previous study, Ficarra et al. concluded that the prevalence of PPI was affected by patient characteristics, surgeon experience, and surgical technique [12]. The incontinence rates were reported as 4-31% for robotic prostatectomy, 7-40% for open radical prostatectomy, and 5-34% for laparoscopic prostatectomy [13]. When the surgical methods used for benign prostatic hyperplasia (BPH) were compared, the incontinence rates (5-6%) were similar in TURP and Holmium laser enucleation of the prostate [14,15]. The total number of prostatectomies performed for malign and benign conditions increases annually; thus, there is also a substantial increase in the PPI rates.

Previous reports have reported that AUS has excellent success (70-90%) and satisfaction (90%) rates, even in severe incontinence [16]. AUS has the longest follow-up with the greatest level of evidence and is therefore considered the gold standard management of PPI [17]. However, mechanical failure is more common in AUS, requiring revision surgery in up to 30% of patients [18]. In some studies, many sling devices have been shown to have good success rates of 77-92% [16,19]. However, the average cure and improvement rates are published as 60-77% and 70-90%, respectively after adjustable male sling implantation [17]. When patients with moderate PPI are informed about the pros and cons of these two techniques, most prefer male sling surgery. In addition, 25% of patients with severe PPI prefer male sling surgery despite their physician’s recommendation of AUS [5]. The outcomes of published articles may be biased by patient characteristics or the definition of treatment success.

The most reliable method to determine whether a treatment result is successful is the measurement of the number and weight of pads required by patients per day. We used the terms ‘cured’ and ‘improved’ to define a successful treatment since these criteria had been used by two large studies for the surgical technique [9,10]. In the present study, the number and weight of pads per day were decreased at the last follow-up visit compared to the preoperative data although none of the patients had mild incontinence (p = 0.001 and p = 0.001, respectively). In the literature, the reduced number of pad use is given as 3.3 to 3.6 [9,10]. In addition, in their cohort, Aagaard et al. reported that the pad weight was reduced to 250 gr and the number of pads was reduced to four per day [20]. Our results were found compatible with the literature.

The symptoms of urinary incontinence and its impact on quality of life are classically evaluated by taking patients’ medical histories. However, clinicians also need a tool to assess these conditions objectively. For this purpose, we used a validated form of the ICIQ-SF questionnaire, which is widely adopted as a reliable and reproducible form. In our study, the ICIQ -SF score statistically significantly decreased compared to the baseline scores (p < 0.001). In a small-size study with ArgusT, the ICIQ-SF score decreased from 17.3 ± 2.8 to 2.4 ± 3.8 at the postoperative sixth-month follow-up [21]. In another study that assessed the outcome of the AdVance sling, the authors stated that the ICIQ-SF score decreased from a baseline of 17.7 to 8.0 [22]. Similarly, Bauer et al. used ICIQ-SF to evaluate the outcome of AdVanceXP and found a 10.8-point decrease (14.9 to 4.1) between the baseline value and the 36th-month follow-up [23]. Our results are in agreement with the literature, and we believe that ICIQ is a reliable questionnaire to assess the outcome.

In our study, the etiology of PPI was TURP in eight patients and RP in the remaining eight. We found that the success rate was not affected by etiology, and the Argus sling was equally effective in the treatment of incontinence following TURP and RP (p = 0.50). In other studies, the rate of TUR-P varied between 9.9% and 14.6%, but to the best of our knowledge, none of those studies compared the outcomes in terms of the patients who developed incontinence after TURP and RP [18,20].

The outcomes of male slings may be inversely proportional to the preoperative conditions. Several studies evaluated the factors associated with outcomes and concluded that the severity of baseline incontinence might affect the outcome. Determining the severity of urinary incontinence is important in deciding on the management of this condition, and it can be categorized according to the number and weight of pads per day [24]. Collaro et al. reported that with each 1-g increase in the preoperative 24-hour pad weight, there was an estimated risk of a 0.4% decrease in the cure rate [25]. From another point of view, in two separate studies using the AdVance sling, the 24-hour pad weight of over 200 mg was determined as a factor that increased the risk of failure [26,27]. In our study, when we evaluated the association of the baseline characteristics with the outcomes of the Argus sling, we did not see any statistically significant difference in pads/day, 24-hour pad weight, and ICIQ-SF score according to the outcomes, although these parameters were higher in the failure group. In addition, when we re-analyzed the groups as above and below 200 gr pad weight, there was no statistically significant result (p > 0.05). These findings may be due to the low number of cases in our study, which did not provide sufficient evidence to compare pre-operative factors that affected the success and failure of treatment.

Postoperative satisfaction levels and the recovery status of patients are perhaps the most important goals of urinary incontinence surgery. In addition to objective data, such as the number of pads used and 24-hour ped test weight, it is important for patients to evaluate this situation from their own perspective. To this end, many questionnaires have been developed. Bochove et al. reported patient satisfaction with the patient global impression of improvement scale consisting of a single question and showed that 84% of patients thought their condition had improved [10]. Siracusano, who performed a similar assessment, additionally found that the reduction in the number of pads was associated with higher patient satisfaction (p < 0.001) [9]. In this study, we used a questionnaire we routinely utilize in our clinic, which comprises three questions with three possible responses to evaluate patient satisfaction, and found that the patients with unfavorable outcomes were more dissatisfied. As in the literature, we consider that objective and subjective evaluations offer similar results. The most reported complications of male sling surgery include infection, erosion, obstruction, chronic perineal pain, and paresthesia. In a systematic review, the overall complication rate of male sling surgery was found to be 12.3% [13]. In our study, we observed Clavien-Dindo grade IIIa complications in three (18.8%) cases, which were all successfully managed under spinal anesthesia (by endoscopic stricturotomy in two and surgical debridement with primary wound closure in one). Due to the Argus male sling having silicone arms, unlike Prolene mesh, it does not result in a tissue reaction that leads to the development of fibrosis. For this reason, it is also easier to remove than Prolene mesh. However, when the male sling is removed, it loses its effect because there is no fibrosis in the adjacent tissue. In our patient with skin erosion, surgical debridement, and primary wound closure were sufficient to achieve normal healing.

This study has several limitations, such as the single-center design and the relatively small number of patients. Another limitation is that we did not measure patients' expectations before the operation, and we only assessed patient satisfaction after the operation. If a patient had a low expectations concerning surgery, even minimal improvement could meet his expectation. Further multi-center studies with a larger number of patients are required.

## Conclusions

In conclusion, male sling surgery can be safely performed in patients with moderate to severe stress urinary incontinence, with low complications and high success rates. No differences could be identified between functional outcomes due to the etiology of stress urinary incontinence (TURP vs. RP) which may imply an advantage for adjustability.
